# Ophthalmia and Astigmatism

**Published:** 1897-12

**Authors:** Levi Hoopes

**Affiliations:** West Chester, Pa.


### OPHTHALMIA AND ASTIGMATISM.

LEVI HOOPES, M. D., WEST CHESTER, PA.

Jas. B. H——, aged 11 1-2 years, light complexion, rather small for his age, came to me suffering from opthalmia, with which he had been troubled for about two years, and most of that time had been under old-school treatment, with but little benefit. The margins of the lids were inflamed and agglutinated at night. They were full of the so-called "wild hairs," there being scarcely a healthy hair in the lashes. He complained of a sensation of sand in the eyes; photophobia so that he could scarcely look up, but kept his eyes on the ground or floor, but it was somewhat better in the open air, so that he desired to be out of doors. Reading made his eyes water, and then he wanted to rub them so he could see better. Vision was about $\frac{2}{3}$ in each eye. Astigmatism vertical in right eye and horizontal in left. I gave three doses of Puls. 2c., which was followed by improvement for about three weeks when it seemed to come to a standstill; I then gave three doses of Puls. M (Finke) after which the improvement again proceeded for about three weeks when all the symptoms appeared to be removed except the myopia, which was somewhat improved, and the astigmatism, which remained the same. I had about concluded that these conditions would have to be corrected with glasses, but the patient was so strongly opposed to wearing them that I concluded to try the effect of Physostigma 30 on him; I therefore gave him three doses of the remedy, and he returned in a week with normal vision, the astigmatism having all disappeared.

This case goes to show that much may be done with homœopathic remedies in many cases toward correcting visual defects, more especially where they arise from constitutional conditions and not from defective formation of the eye itself. Of course the latter condition can only be corrected by proper glasses; but I think homœopathic physicians should first exhaust their therapeutic means before consigning such cases to the optician.

Last spring I cured another case in a child about the same age, with three doses of Gels. 2c., whose parents had been told by an allopathic physician that glasses were the only remedy.

